# Cigarette smoking slows the on- and the off- cardiorespiratory and gas-exchange kinetics during moderate exercise in young, physically active adults

**DOI:** 10.1007/s00421-026-06150-8

**Published:** 2026-03-09

**Authors:** Marta Borrelli, Asia Motalli, Christian Doria, Nicholas Toninelli, Stefano Longo, Giuseppe Coratella, Emiliano Cè, Susanna Rampichini, Fabio Esposito

**Affiliations:** Department of Biomedical Sciences for Health, Università degli Studi di Milano, Via Giuseppe Colombo 71, Milan, 20133 Italy

**Keywords:** Cycle ergometer, Heart rate, Oxygen uptake, Recovery, Smoker

## Abstract

**Purpose:**

Cigarette smoking (CS) impact on cardiopulmonary function has been extensively investigated on sedentary, middle-aged smokers (SMK) with pulmonary disease, but not on young SMK with high fitness level. This study evaluated the cardiopulmonary and gas-exchange kinetics during and after moderate exercise in young, physically active SM without known diseases.

**Methods:**

Ten SMK (age: 21 ± 2 year., body mass: 78 ± 6 kg; stature: 1.79 ± 0.07 m; 12 ± 5 cigarette/day for 6 ± 2 year.; mean ± SD) and twelve non-smokers (CTRL; age: 24 ± 3 year., body mass: 78 ± 9 kg; stature: 1.80 ± 0.08 m) matched also for exercise habits performed an incremental cycloergometric test to assess maximum pulmonary oxygen uptake ($\:{\dot{\mathrm{V}}}_{{\mathrm{O}}_{2}\:\mathrm{m}\mathrm{a}\mathrm{x}}$) and first ventilatory threshold (VT_1_). After pulmonary evaluation, participants performed four 6-min moderate-intensity tests at 90% VT_1_. The time constant (τ) of the on- and off-phases were determined for expiratory ventilation ($\:{\dot{\mathrm{V}}}_{\mathrm{E}}$), $\:{\dot{\mathrm{V}}}_{{\mathrm{O}}_{2}}$, heart rate (*f*_H_) and cardiac output ($\:\dot{\mathrm{Q}}$).

**Results:**

Despite similar static lung volumes, SMK exhibited lower peak expiratory flow (-21%; *P* = 0.009) and maximal voluntary ventilation (-12%; *P* = 0.008). SMK had lower $\:{\dot{\mathrm{V}}}_{{\mathrm{O}}_{2\:\mathrm{m}\mathrm{a}\mathrm{x}}}$ (3657 ± 325 vs. 3397 ± 316 ml∙min^− 1^ for CTRL and SMK, respectively; *P* = 0.009) and mechanical power at VT_1_ (201 ± 26 vs. 185 ± 16 W for CTRL and SMK respectively; *P* = 0.041). In on-phase, SMK demonstrated longer τ in $\:\dot{\mathrm{Q}}$ (+ 22%; *P* = 0.032), $\:{f}_{\mathrm{H}}$ (+ 56%; *P* = 0.005), $\:{\dot{\mathrm{V}}}_{{\mathrm{O}}_{2}}$ (+ 41%; *P* = 0.032), $\:{\dot{\mathrm{V}}}_{\mathrm{E}}$ (+ 47%; *P* = 0.007) and. In off-phase, τ in SMK was lengthened for $\:\dot{\mathrm{Q}}$ (+ 51%; *P* = 0.041), $\:{f}_{\mathrm{H}}$ (+ 42%; *P* = 0.022), $\:{\dot{\mathrm{V}}}_{{\mathrm{O}}_{2}}$ (+ 20%; *P* = 0.002) and $\:{\dot{\mathrm{V}}}_{\mathrm{E}}$ (+ 42%; *P* = 0.018).

**Conclusion:**

CS slowed cardiopulmonary and gas-exchange kinetics at moderate exercise even in young individuals with short smoking history.

## Introduction

Cigarette smoking (CS) is one of the most impactful risk factors for cardiovascular disease and the leading preventable cause of death (World Health Organization 2017). The cigarette compounds, indeed, negatively affect different apparatus, among which are the cardiopulmonary and muscular systems both at rest and during exercise (Regan et al. 2015; de Tarso Muller et al. 2019). Smoke-produced carbon monoxide (CO) binds to haemoglobin (HbCO) reducing the O_2_ delivery (McDonough and Moffatt 1999; de Tarso Muller et al. 2019); nicotine stimulates the sympathetic nervous system, increasing resting heart rate ($\:{f}_{\mathrm{H}\:}$), cardiac work and peripheral vasoconstriction (Papathanasiou et al. 2007); tar produced by tobacco combustion increases pulmonary airway resistance and the work of breathing (Rotstein et al. 1991; McDonough and Moffatt 1999). Additionally, reactive oxygen species and other oxidants in cigarettes, which contribute to an inflammatory state, impair skeletal muscle fibres function, especially at mitochondrial level (Neves et al. 2016).

CS has been shown to alter cardiopulmonary, muscular, and metabolic responses, leading to reduced exercise intolerance (de Tarso Muller et al. 2019). Specifically, some studies have demonstrated a decline in exercise capacity during submaximal work rate, evidenced by higher $\:{f}_{\mathrm{H}\:}$ (Rotstein et al. 1991; Papathanasiou et al. 2007; Mendonca et al. 2011), reduced gas exchange (i.e., increased ventilatory equivalent for oxygen, $\:{\dot{\mathrm{V}}}_{\mathrm{E}}/{\dot{\mathrm{V}}}_{{\mathrm{O}}_{2}}$ and for carbon dioxide, $\:{\dot{\mathrm{V}}}_{\mathrm{E}}/{\dot{\mathrm{V}}}_{{\mathrm{C}\mathrm{O}}_{2}}$) (Sven et al. 2010) and increased blood lactate concentration ([La^−^]) at the same work rate (Rotstein et al. 1991; Sven et al. 2010).

Numerous studies have reported a link between CS and lower maximum pulmonary oxygen uptake ($\:{\dot{\mathrm{V}}}_{{\mathrm{O}}_{2}\mathrm{m}\mathrm{a}\mathrm{x}}$) and $\:{f}_{\mathrm{H}\:\mathrm{m}\mathrm{a}\mathrm{x}}$ in sedentary, middle-aged smokers (SMK) with (Sven et al. 2010; Elbehairy et al. 2017) and without chronic obstructive pulmonary disease (COPD) (Elbehairy et al. 2016; Sadaka et al. 2021). To date, limited attention has been given to young, physically active SMK at the early stage of smoking history without known lung or cardiovascular disease. This condition is particularly relevant given that, globally, smoking initiation occurs between the ages of 15 and 24 (Reitsma et al. 2021). Moreover, approximately 20% of COPD patients aged ≥ 40 years report having started smoking during childhood (Sargent et al. 2023). Previous studies on this young asymptomatic population have some major methodological flaws (Chevalier et al. 1963; Rotstein et al. 1991; Bernaards et al. 2003; Mendonca et al. 2011; Lorensia et al. 2021; Borrelli et al. 2025). One study, indeed, lacked a control group (Mendonca et al. 2011), others failed to match SMK to controls by exercise habits (Chevalier et al. 1963; Bernaards et al. 2003), while another focused only on lung function assessment (Lorensia et al. 2021). Our previous study (Borrelli et al. 2025), after matching SMK and control group for age and exercise habits, reported lower $\:{\dot{\mathrm{V}}}_{{\mathrm{O}}_{2}}$ and expiratory ventilation ($\:{\dot{\mathrm{V}}}_{\mathrm{E}}$) at peak incremental exercise, with no differences between SM and controls at submaximal level, and noted slower cardiopulmonary and gas-exchange kinetics during the recovery phase. However, in all these studies there has been limited investigation into the potential effects of CS on cardiopulmonary and gas-exchange response during moderate-intensity exercise and the subsequent recovery phase. Square wave exercise tests at moderate-intensity (i.e., below the onset of lactate accumulation threshold) enable the evaluation of the cardiopulmonary and gas-exchange kinetics, providing an index associated with the aerobic performance and exercise tolerance (Poole and Jones 2012). The rest-to-exercise (on-phase) and exercise-to-rest transition upon exercise cessation (off-phase) provide valuable insight into muscular energetics and mitochondrial function (di Prampero 1981; Poole and Jones 2012; Ferretti 2015; Ferretti et al. 2022; Rossiter and Poole 2024) without necessitating the attainment of exhaustion, as required in maximal tests. During moderate-intensity whole-body exercise, muscle $\:{\dot{\mathrm{V}}}_{{\mathrm{O}}_{2}}$ on-phase is represented by the time constant of phase II pulmonary $\:{\dot{\mathrm{V}}}_{{\mathrm{O}}_{2}}$ kinetics, which corresponds to that of phosphocreatine breakdown (Binzoni et al. 1992; Ferretti et al. 2022).

Only two studies focused on the CS impact on the cardiopulmonary and gas-exchange response to exercise in SMK (Chevalier et al. 1963; Rotstein et al. 1991). Among these, one study (Rotstein et al. 1991) found slower cardiopulmonary kinetics following the acute consumption of three cigarettes immediately before testing, compared to a 24-hour smoking washout period. However, this investigation lacked a control group and did not analyse the off-phase kinetics. The other study (Chevalier et al. 1963) examined the cardiopulmonary and gas-exchange kinetics during moderate-intensity exercise in young, sedentary SMK. Nevertheless, this study assessed only $\:{\dot{\mathrm{V}}}_{{\mathrm{O}}_{2}}$ and $\:{f}_{\mathrm{H}\:}$, applying an outdated methodological approach for kinetics analysis. Specifically, this investigation did not utilize a breath-by-breath system, and the cardiopulmonary and gas-exchange kinetics were not modelled with an exponential function, reporting only the values at the third and the fifth minute of exercise and of recovery.

Hence, this study sought to assess the CS effects on cardiopulmonary and gas-exchange phase II kinetics during and after moderate-intensity exercise in young, physically active SMK without known lung or cardiovascular disease. We hypothesized that, despite their age, brief smoking history and good fitness level, which could have counterbalanced the harmful CS effects, SMK would exhibit slower kinetics.

## Materials and methods

### Participants

Based on pilot testing and our previous work (Borrelli et al. 2025), the optimal sample size was computed using a statistical software (G-Power 3.1, Dusseldorf, Germany), expecting a large Cohen’s d effect size (1.3) in τ differences between groups and applying a two-tailed unpaired Student’s t-test. Considering a required power (1 − β) > 0.80 and an α < 0.05, the desired sample size was 22 participants. Therefore, ten young, physically active male SMK (number of cigarettes per day: 12 ± 5; history of smoking; 6 ± 2 years; cigarette exposure: 3.2 ± 1.7 pack-years) and twelve male non-smokers (CTRL), matched for age and exercise habits, completed the protocol (International Physical Activity Questionnaire, IPAQ; 3983 ± 1670 vs. 4414 ± 1757 METs min-1·week-1; *P* = 0.565). The current dataset includes part of the participants from our previously published work (Borrelli et al. 2025), with the addition of other SMK and CTRL who were enrolled at a later stage. Table 2 reported the anthropometric characteristics. The inclusion criteria for CS were smoking at least 6 cigarettes per day for a minimum of two continuous years (Okuyemi et al. 2002). The exclusion criteria for both groups were: (i) cardiovascular and pulmonary diseases; (ii) musculoskeletal impairments; and (iii) medications altering cardiovascular and pulmonary responses. 

Participants were fully informed about the study’s purpose and the experimental design and gave written consent to participate. The study conformed to the Declaration of Helsinki and was approved by the local ethics committee (#77/20).

### Experimental procedures

All experimental sessions were conducted in a climate-controlled laboratory (constant temperature of 20 ± 1 °C and relative humidity of 50 ± 5%) at approximately the same time of the day to minimize bias induced by circadian rhythms.

Participants reported to the laboratory four times, separated by at least 48 h. Each day of testing, participants were asked to abstain from caffeine and any other stimulant substances for at least 12 h, and to refrain from heavy exercise for at least 24 h prior the tests. SMK were instructed to smoke the last cigarette 1.5 h before the test to allow 5–16% elimination of blood HbCO to avoid the acute effects of CS (McDonough and Moffatt 1999).

### Familiarization, anthropometric and pulmonary function assessment

In the first session, the participants familiarized with the equipment used for cardiopulmonary testing. Body mass and stature were measured using a mechanical scale with a stadiometer (Asimed, Samadell, Barcelona). On the same day, pulmonary function was assessed by a portable spirometer (Pony Fx, Cosmed, Rome, Italy) according to the following guidelines (Graham et al. 2019). In particular, vital capacity, dynamic lung volumes and instantaneous expiratory flow parameters throughout the manoeuvre were measured. Maximal inspiratory and expiratory pressure (MIP and MEP, respectively) were assessed at the mouth using a portable manometer equipped with a mouthpiece. Predicted values were determined according to Miller et al. (2005). After familiarization with the procedure, participants repeated the manoeuvre three times, and the highest value was considered.

### Incremental exercise test

On the second day, $\:{\dot{\mathrm{V}}}_{{\mathrm{O}}_{2}\mathrm{m}\mathrm{a}\mathrm{x}}$ and $\:{\dot{\mathrm{W}}}_{\mathrm{m}\mathrm{a}\mathrm{x}}$ as well as the first and the second ventilatory thresholds ($\:{\mathrm{V}\mathrm{T}}_{1}$ and $\:{\mathrm{V}\mathrm{T}}_{2}$, respectively) were assessed by a step-wise incremental test as reported in a previous study of our group (Borrelli et al. 2025). [La^−^] was determined at rest, at the end of each work rate and at minute 1, 3 and 5 of recovery to assess the [La^−^] at peak. At the same time, participants were asked to indicate their rate of perceived exertion (RPE) on a general (RPE_GEN_; Borg 6–20), muscular and pulmonary (RPE_MUSC_ and RPE_RESP_, respectively; CR-10) level.

### Kinetics assessment

During both the third and the fourth visits, participants performed two square wave transitions to a moderate-intensity work rate that elicited a $\:{\dot{\mathrm{V}}}_{{\mathrm{O}}_{2}}$ corresponding to 90% of $\:{\mathrm{V}\mathrm{T}}_{1}$ assessed during the first session. Participants completed four trials each including cycling 6 min at 20 W and 6 min at 90% $\:{\mathrm{V}\mathrm{T}}_{1}$. Each trial was separated by a 30-min resting recovery (Murias et al. 2011). Participants were asked to maintain the pedalling rate between 60 and 70 rpm. [La^−^] was determined at rest, at the 4^th^ and the 6^th^ minute of the first step transition.

### Measurements

Tests were performed on an electro-mechanically braked cycle ergometer (mod. 839E, Monark, Sweden). During the experiments, the work rate and cadence were continuously recorded. $\:{\dot{\mathrm{V}}}_{\mathrm{E}}$, $\:{\dot{\mathrm{V}}}_{{\mathrm{O}}_{2}}$, pulmonary frequency ($\:{f}_{\mathrm{R}}$), tidal volume (V_T_) and carbon dioxide production ($\:{\dot{\mathrm{V}}}_{{\mathrm{C}\mathrm{O}}_{2}}$) were measured on a breath-by-breath basis by a metabolic unit that was calibrated before each test (Quark b^2^, Cosmed, Rome, Italy). Moreover, $\:{\dot{\mathrm{V}}}_{\mathrm{E}}/{\dot{\mathrm{V}}}_{{\mathrm{O}}_{2}}$ and $\:{\dot{\mathrm{V}}}_{\mathrm{E}}/{\dot{\mathrm{V}}}_{{\mathrm{C}\mathrm{O}}_{2}}$, end-tidal oxygen pressure ($\:{\mathrm{P}\mathrm{e}\mathrm{t}\mathrm{O}}_{2}$), end-tidal carbon dioxide pressure ($\:{\mathrm{P}\mathrm{e}\mathrm{t}\mathrm{C}\mathrm{O}}_{2}$) and respiratory exchange ratio (RER) were calculated. $\:{f}_{\mathrm{H}}$ and cardiac output ($\:\dot{\mathrm{Q}})$ were acquired by a non-invasive hemodynamic monitor based on impedance cardiography (PhysioFlow^®^ Imped monitor, Manatec Biomedical, Paris, France). Lastly, 20 µl arterialized blood samples were collected from the ear lobe and analysed by an enzymatic-amperometric system (Labtrend, Bio Sensor Technology GmbH, Berlin, Germany) to determine [La^−^].

### Data analysis

All data were analysed off-line. The pulmonary and gas exchange responses were edited of spurious breaths, by deleting values outside three standard deviation (SD) from the local mean (Lamarra et al. 1987).

$\:{\dot{\mathrm{V}}}_{{\mathrm{O}}_{2}\mathrm{m}\mathrm{a}\mathrm{x}}$ was determined as the value obtained from the plateau in the relationship between $\:{\dot{\mathrm{V}}}_{{\mathrm{O}}_{2}}$ and $\:\dot{\mathrm{W}}$ during the incremental step-wise test. In the event that the plateau did not occur, subsidiary criteria for definition of $\:{\dot{\mathrm{V}}}_{{\mathrm{O}}_{2}\mathrm{m}\mathrm{a}\mathrm{x}}$ were utilized (Åstrand et al. 2003; Ferretti 2014). The $\:{\dot{\mathrm{V}}}_{{\mathrm{O}}_{2}}$ value corresponding $\:{\mathrm{V}\mathrm{T}}_{1}$ was defined by three experienced operators as $\:{\dot{\mathrm{V}}}_{{\mathrm{O}}_{2}}$ at which $\:{\dot{\mathrm{V}}}_{\mathrm{E}}/{\dot{\mathrm{V}}}_{{\mathrm{O}}_{2}}$ and $\:{\mathrm{P}\mathrm{e}\mathrm{t}\mathrm{O}}_{2}$ increased with time with no concomitant changes in $\:{\dot{\mathrm{V}}}_{\mathrm{E}}/{\dot{\mathrm{V}}}_{\mathrm{C}{\mathrm{O}}_{2}}$ and in $\:{\mathrm{P}\mathrm{e}\mathrm{t}\mathrm{C}\mathrm{O}}_{2}$ (Beaver et al. 1986).

For the kinetic analyses, the data of each transition were linearly interpolated to 1-s intervals and time aligned such that time 0 represented the onset of exercise. Only for the on- phase, phase 1 was excluded by visual inspection of the second-by-second data (Murias et al. 2011). All the transients of both the on- and off- phase, modelled from − 180 s to 360 s of the step transition with the assurance that steady-state had been attained, were averaged together and fit by a mono-exponential of this form (Benson et al. 2017):

1 $$\:\mathrm{Y}\left(\mathrm{t}\right)=\:{\mathrm{Y}}_{0}+\mathrm{A}\mathrm{M}\mathrm{P}\:[1-{\mathrm{e}}^{\frac{-(\mathrm{t}-{\mathrm{t}}_{\mathrm{D}})}{{\uptau\:}}}]$$

where, $\:{\mathrm{Y}}_{0}$ constitutes the value of the cardiopulmonary variables immediately before the transient, AMP is the amplitude of the response, τ is the time necessary to reach the 63% of the amplitude, and t_D_ is time delay of the exponential function. The model parameters were free to vary and were estimated by least-squares non-linear regression (Origin, OriginLab Corp., Northampton, MA, USA).

### Statistical analysis

Descriptive statistics were used to define the study sample characteristics. The Shapiro-Wilk test was applied to check the normal distribution. When normality was not confirmed, a logarithmic transformation was applied. If the transformed data followed a normal distribution, parametric tests were used. Specifically, the differences between the two groups of cardiorespiratory and gas-exchange parameters were detected by unpaired Student’s t-test. When normality was not confirmed the Mann-Whitney U test was applied. The differences between the two groups of cardiopulmonary and gas-exchange parameters were detected by unpaired Student’s t-test. The Hedge’s g effect size with 95% confidence interval (CI_95%_) was also calculated and interpreted as follows: 0.00–0.19: trivial; 0.20–0.59: small; 0.60–1.19: moderate; 1.20–1.99: large; ≥ 2.00: very large (Hopkins et al. 2009). A two-way mixed-model ANOVA for repeated measure checked for differences in [La^−^] between groups over time during the test. For all pairwise multiple comparisons, the Bonferroni’s correction was applied. The ANOVA effect size was evaluated with partial eta squared (pη²). All statistical analyses were performed by using statistical software (IBM SPSS Statistics v. 29, Armonk, NY, USA). The significance level was set at α < 0.05. Results are presented as mean ± standard deviation (SD).

## Results

Table 1 reported the pulmonary function parameters.

**Table 1:** Respiratory function test parameters in smokers (SMK) and control group (CTRL)

	CTRL		SMK	
	Absolute	% Predicted	Absolute	% Predicted
FVC (l)	5.9 ± 0.6	108 ± 7	5.6 ± 0.7	104 ± 13
FEV_1_ (l)	5.0 ± 0.6	109 ± 9	4.7 ± 0.5	102 ± 11
FEV_6_ (l)	5.9 ± 0.7	105 ± 7	5.5 ± 0.7	99 ± 11
FEV_1_/FVC	85 ± 4	102 ± 5	83 ± 9	99 ± 11
FEF 25–75%	5.3 ± 1.0	102 ± 19	4.9 ± 1.3	94 ± 25
MEF75%	9.0 ± 1.6	104 ± 16	7.7 ± 1.9	89 ± 22
MEF50%	5.9 ± 1.5	103 ± 26	5.6 ± 1.4	97 ± 25
MEF25%	2.9 ± 0.7	105 ± 27	2.6 ± 0.8	95 ± 28
FET 100%	5.4 ± 2.2	-	7.1 ± 3.3	-
PEF (l·s^− 1^)	10.7 ± 1.8	104 ± 16	8.4 ± 1.8*	82 ± 17*
SVC (l)	5.5 ± 0.6	98 ± 7	5.4 ± 0.6	96 ± 11
ERV (l)	1.8 ± 0.5	108 ± 30	1.9 ± 0.5	110 ± 27
IRV (l)	2.7 ± 0.6	-	2.3 ± 0.5	-
MVV (l·min^− 1^)	192 ± 22	123 ± 9	168 ± 14*	107 ± 9*
MIP (cmH_2_O)	117 ± 14	106 ± 13	116 ± 22	103 ± 19
MEP (cmH_2_O)	129 ± 24	88 ± 17	127 ± 15	86 ± 10

FVC, forced vital capacity; FEV_1_, forced expiratory volume during the 1^st^ s of the test; FEV_6_, forced expiratory volume during the 6^th^ s of the test; FEF 25–75%, forced expiratory flow at 25 and 75% of the pulmonary volume; MEF75%; instantaneous expiratory flow when 25% of FVC has to be expired; MEF50%, instantaneous expiratory flow when 50% of FVC has to be expired; MEF25%, instantaneous expiratory flow when 75% of FVC has to be expired; FET 100%, forced expiratory time; PEF, peak expiratory flow; SVC, slow vital capacity; ERV, expiratory reserve volume; IRV, inspiratory reserve volume; MVV, maximal voluntary ventilation; MIP, maximal inspiratory mouth pressure; MEP, maximal expiratory mouth pressure. Predicted values were determined according to Miller et al. (2005). Data are shown as mean ± standard deviation (SD). * *P* < 0.05 vs. CTRL

No differences between the two groups emerged in most of the cardiopulmonary and gas-exchange parameters during baseline recordings.

SMK and CTRL exhibited similar $\:{f}_{\mathrm{H}}$ (77 ± 12 vs. 73 ± 8 beats·min^− 1^, respectively), $\:{\dot{\mathrm{V}}}_{{\mathrm{O}}_{2}}$ (329 ± 68 vs. 362 ± 53 ml·min^− 1^, respectively) and $\:{\dot{\mathrm{V}}}_{{\mathrm{C}\mathrm{O}}_{2}}$ (259 ± 83 vs. 311 ± 53 ml·min^− 1^, respectively). Nevertheless, SMK had lower $\:{\dot{\mathrm{V}}}_{\mathrm{E}}$ than CTRL (10.1 ± 2.7 vs. 12.2 ± 1.9 l·min^− 1^, respectively; *P* = 0.044; g = -0.88, moderate; 95% CI = -0.004–1.76). The main outcomes of the incremental test are reported in Table 2.

**Table 2:** Main outcomes of step-wise incremental test at peak of exercise

	CTRL (*n* = 12)	SMK (*n* = 10)
Age (years)	23.5 ± 3.0	21.3 ± 1.9
Body mass (kg)	78.3 ± 9.1	77.7 ± 5.5
Stature (m)	1.80 ± 0.08	1.79 ± 0.07
RER	1.08 ± 0.03	1.17 ± 0.05*
$\:{f}_{\mathrm{H}}$ (beats∙min^− 1^)	184 ± 10	186 ± 9
$\:{\dot{\mathrm{V}}}_{\mathrm{E}}$ (l∙min^− 1^)	141 ± 16	127 ± 12*
$\:{f}_{\mathrm{R}}$ (breaths∙min^− 1^)	57 ± 10	50 ± 7*
$\:{\mathrm{V}}_{\mathrm{T}}$ (l)	2.55 ± 0.45	2.64 ± 0.38
[La^−^] (mM)	9.4 ± 2.0	9.7 ± 1.5
VT_1_ (W)	201 ± 26	185 ± 16*
VT_2_ (W)	252 ± 32	225 ± 20*
VT_1_ (ml∙min^− 1^)	2811 ± 316	2665 ± 285
VT_2_ (ml∙min^− 1^)	3361 ± 387	3102 ± 139
RPE_GEN_ (a.u.)	19 ± 1	19 ± 1
RPE_MUSC_ (a.u.)	10 ± 1	10 ± 1
RPE_RESP_ (a.u.)	10 ± 1	10 ± 1

Maximal mechanical aerobic power ($\:\dot{\mathrm{W}}$), pulmonary oxygen uptake ($\:{\dot{\mathrm{V}}}_{{\mathrm{O}}_{2}}$), carbon dioxide production ($\:{\dot{\mathrm{V}}}_{{\mathrm{C}\mathrm{O}}_{2}}$), respiratory exchange ratio (RER), heart rate ($\:{f}_{\mathrm{H}}$), expiratory ventilation ($\:{\dot{\mathrm{V}}}_{\mathrm{E}}$), respiratory rate ($\:{f}_{\mathrm{R}}$), tidal volume ($\:{\mathrm{V}}_{\mathrm{T}}$), blood lactate concentration ([La^−^]), first and second ventilatory thresholds (VT_1_ and VT_2_, respectively) rates of perceived exertion on a general (RPE_GEN_; Borg 6–20), respiratory and muscular (RPE_MUSC_ and RPE_RESP_; CR-10). Data are shown as mean ± standard deviation (SD). * *P* < 0.05 vs. CTRL

Regarding the kinetics during moderate exercise, normality was not confirmed for τ and Y_0_ of $\:{\dot{\mathrm{V}}}_{\mathrm{E}}$ and Y_0_ of $\:{\dot{\mathrm{V}}}_{{\mathrm{C}\mathrm{O}}_{2}}$ during off-phase. Therefore, logarithmic transformation was applied, yielding normal distributions, and parametric tests were used. In contrast, for AMP of $\:{\dot{\mathrm{V}}}_{{\mathrm{O}}_{2}}$ and $\:{\dot{\mathrm{V}}}_{{\mathrm{C}\mathrm{O}}_{2}}$ during on-phase and AMP of $\:{\dot{\mathrm{V}}}_{{\mathrm{C}\mathrm{O}}_{2}}$ during off-phase, normality was not confirmed, requiring the Mann-Whitney U test.

**Fig. 1:**
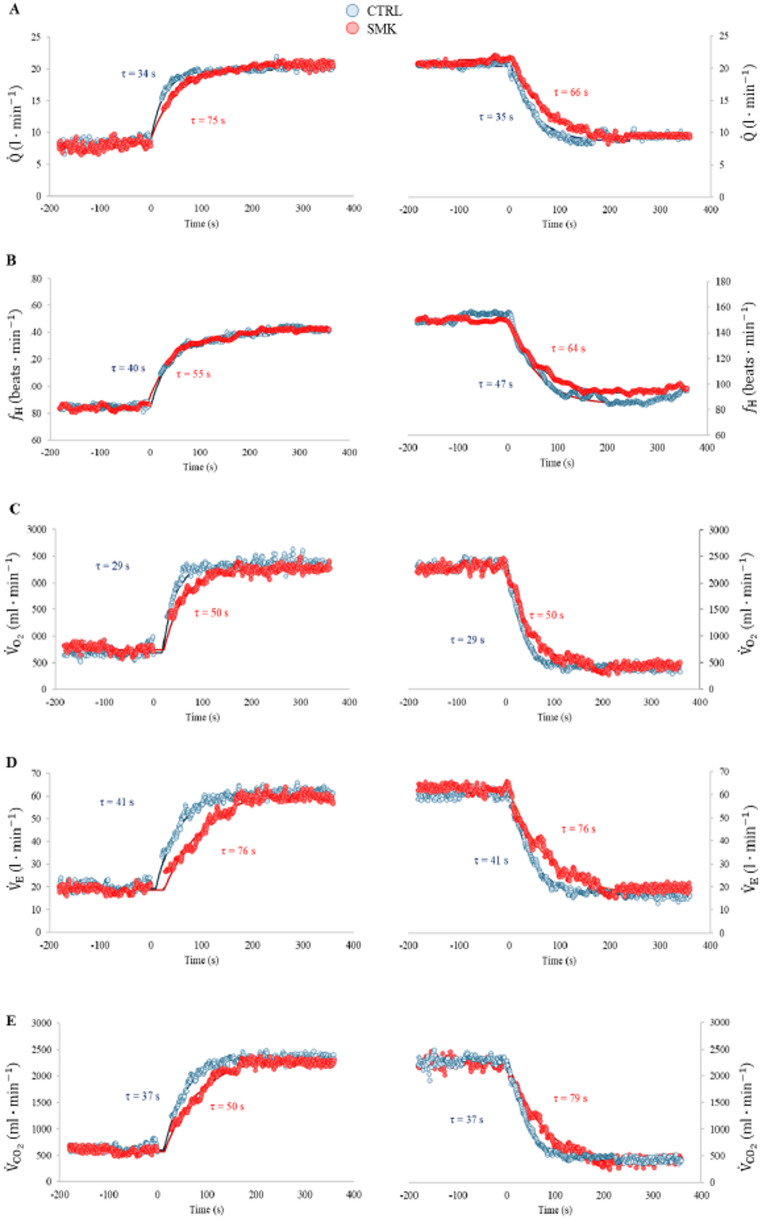
Cardiopulmonary and gas-exchange on- and off-response to moderate-intensity square wave exercise by a representative subject of smoker (black circle) and control group (white circle). Cardiac output (Q̇, panel A); heart rate $\:($*f*_H_, panel B); expiratory ventilation $\:({\dot{\mathrm{V}}}_{\mathrm{E}},$ panel C), pulmonary oxygen uptake $\:({\dot{\mathrm{V}}}_{{\mathrm{O}}_{2}}$, panel D) and carbon dioxide production $\:({\dot{\mathrm{V}}}_{{\mathrm{C}\mathrm{O}}_{2}},$ panel E)

The cardiopulmonary and gas-exchange response to moderate-intensity square wave exercise during the on- and off-phase in representative participants has been illustrated in Fig. 1.

During the on-phase, SM revealed comparable Y_0_ to CTRL of $\:\dot{\mathrm{Q}}$ (8 ± 1 vs. 8 ± 1 l·min^− 1^ for SM and CTRL, respectively), $\:{f}_{\mathrm{H}}$ (89 ± 12 vs. 81 ± 9 beats·min^− 1^ for SM and CTRL, respectively), $\:{\dot{\mathrm{V}}}_{{\mathrm{O}}_{2}}$ (680 ± 225 vs. 736 ± 108 ml·min^− 1^ for SM and CTRL, respectively), $\:{\dot{\mathrm{V}}}_{\mathrm{E}}$ (18 ± 5 vs. 19 ± 2 l·min^− 1^ for SM and CTRL, respectively) and $\:{\dot{\mathrm{V}}}_{{\mathrm{C}\mathrm{O}}_{2}}$ (566 ± 184 vs. 616 ± 106 ml·min^− 1^ for SM and CTRL, respectively). Similarly, during the off-phase, SM had similar Y_0_ compared to CTRL of $\:\dot{\mathrm{Q}}$ (19 ± 2 vs. 18 ± 2 l·min^− 1^ for SM and CTRL, respectively), $\:{f}_{\mathrm{H}}$ (137 ± 15 vs. 137 ± 13 beats·min^− 1^ for SM and CTRL, respectively), $\:{\dot{\mathrm{V}}}_{{\mathrm{O}}_{2}}$ (2277 ± 295 vs. 2500 ± 270 ml·min^− 1^ for SM and CTRL, respectively), $\:{\dot{\mathrm{V}}}_{\mathrm{E}}$ (56 ± 7 vs. 61 ± 7 l·min^− 1^ for SM and CTRL, respectively) and $\:{\dot{\mathrm{V}}}_{{\mathrm{C}\mathrm{O}}_{2}}$ (2209 ± 275 vs. 2413 ± 258 ml·min^− 1^ for SM and CTRL, respectively).

No differences between SM and CTRL were found in the AMP of all the parameters. Specifically, during the on-phase, SM exhibited similar AMP compared to CTRL of $\:\dot{\mathrm{Q}}$ (9 ± 2 vs. 9 ± 2 l·min^− 1^ for SM and CTRL, respectively), $\:{f}_{\mathrm{H}}$ (53 ± 12 vs. 56 ± 10 beats·min⁻¹ for SM and CTRL, respectively), $\:{\dot{\mathrm{V}}}_{{\mathrm{O}}_{2}}$ (1684 ± 219 vs. 1745 ± 301 ml·min⁻¹ for SM and CTRL, respectively), $\:{\dot{\mathrm{V}}}_{\mathrm{E}}$ (40 ± 4 vs. 42 ± 7 l·min^− 1^ for SM and CTRL, respectively) and $\:{\dot{\mathrm{V}}}_{{\mathrm{C}\mathrm{O}}_{2}}$ (1744 ± 190 vs. 1809 ± 306 ml·min^− 1^ for SM and CTRL, respectively). During the off- phase, similar AMP results were obtained for SM and CTRL of $\:\dot{\mathrm{Q}}$ (10 ± 2 vs. 9 ± 2 l·min^− 1^ for SM and CTRL, respectively), $\:{f}_{\mathrm{H}}$ (50 ± 12 vs. 54 ± 10 beats·min⁻¹ for SM and CTRL, respectively), $\:{\dot{\mathrm{V}}}_{{\mathrm{O}}_{2}}$ (1881 ± 305 vs. 2110 ± 236 ml·min⁻¹ for SM and CTRL, respectively), $\:{\dot{\mathrm{V}}}_{\mathrm{E}}$ (43 ± 6 vs. 46 ± 6 l·min^− 1^ for SM and CTRL, respectively) and $\:{\dot{\mathrm{V}}}_{{\mathrm{C}\mathrm{O}}_{2}}$ (1877 ± 246 vs. 2036 ± 224 ml·min^− 1^ for SM and CTRL, respectively).

**Fig. 2:**
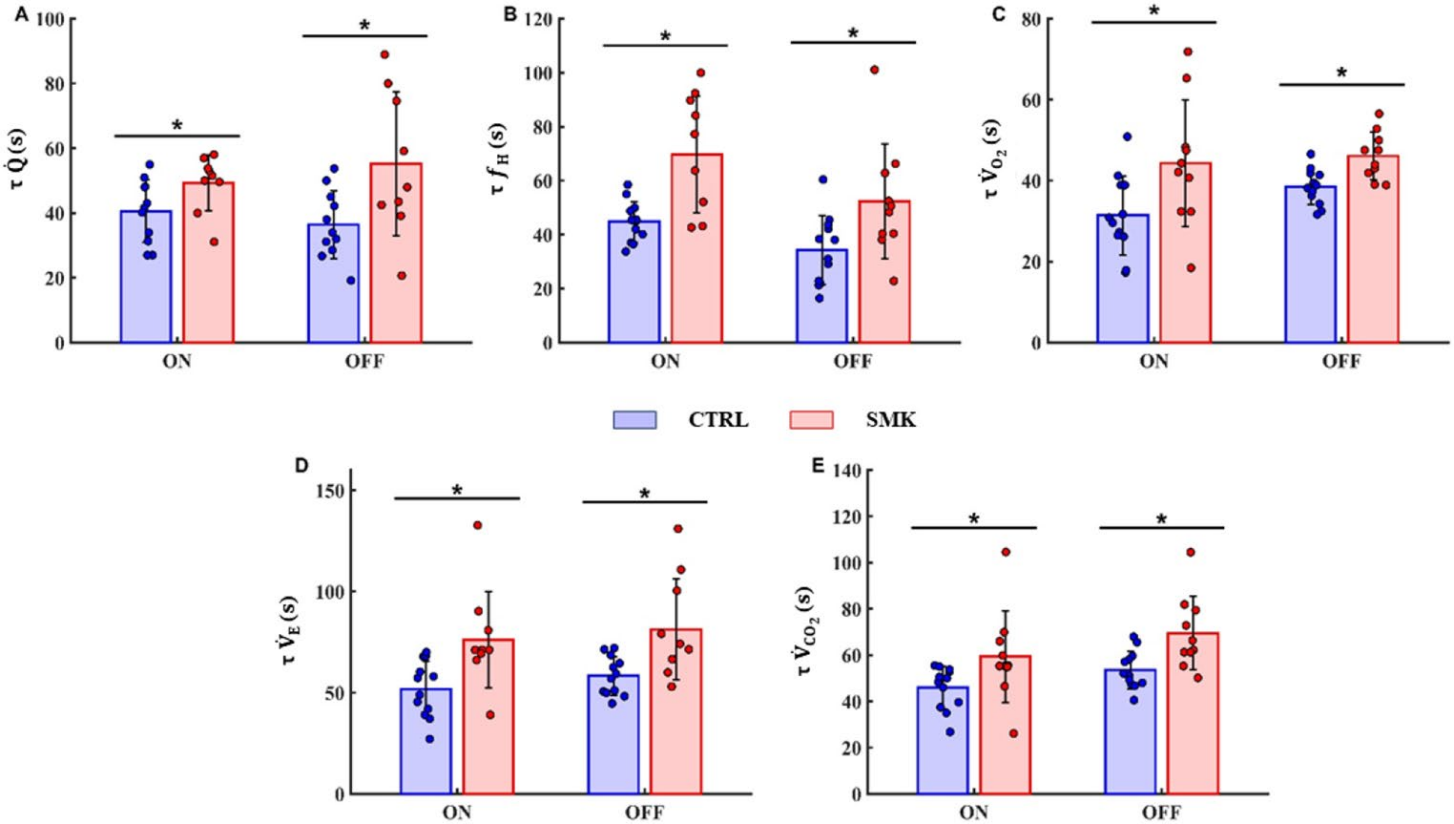
Tau values (τ) of the cardiopulmonary and gas-exchange on- and off-response to moderate-intensity square wave exercise of smoker (SMK, red bars) and control group (CTRL, blue bars). Cardiac output (Q̇, panel A); heart rate $\:($*f*_H_, panel B); expiratory ventilation $\:({\dot{\mathrm{V}}}_{\mathrm{E}},$ panel C), pulmonary oxygen uptake $\:({\dot{\mathrm{V}}}_{{\mathrm{O}}_{2}}$, panel D) and carbon dioxide production $\:({\dot{\mathrm{V}}}_{{\mathrm{C}\mathrm{O}}_{2}},$ panel E)

As reported in Fig. 2, during the on-phase, SM exhibited longer τ compared to CTRL in $\:\dot{\mathrm{Q}}$ (+ 22%; *P* = 0.032; g = -1.09, moderate; CI_95%_ = 0.09–2.05, panel A), $\:{f}_{\mathrm{H}}$ (+ 56%; *P* = 0.005; g = -1.61, large; CI_95%_= 0.62–2.57, panel B), $\:{\dot{\mathrm{V}}}_{{\mathrm{O}}_{2}}$ (+ 41%; *P* = 0.032; g = -0.99, moderate; CI_95%_ = 0.81–1.87, panel C), $\:{\dot{\mathrm{V}}}_{\mathrm{E}}$ (+ 47%; *P* = 0.007; g = -1.29, large; CI_95%_= 0.34–2.12, panel D) and $\:{\dot{\mathrm{V}}}_{{\mathrm{C}\mathrm{O}}_{2}}$ (+ 30%; *P* = 0.047; g = -0.91, moderate; CI_95%_ = -0.01–1.78, panel E). Moreover, during the off-phase, SM presented longer τ compared to CTRL in $\:\dot{\mathrm{Q}}$ (+ 51%; *P* = 0.041; g = -1.12, moderate; CI_95%_ = 0.15–2.05, panel A), $\:{f}_{\mathrm{H}}$ (+ 42%; *P* = 0.022; g = -1.06, moderate; CI_95%_ = 0.15–1.95, panel B), $\:{\dot{\mathrm{V}}}_{{\mathrm{O}}_{2}}$ (+ 20%, respectively *P* = 0.002; g = -1.53, large; CI_95%_ = 0.55–2.47, panel C), $\:{\dot{\mathrm{V}}}_{\mathrm{E}}$ (+ 42%; *P* = 0.018; g = -1.27, large; CI_95%_ = 0.33–2.19, panel D) and $\:{\dot{\mathrm{V}}}_{{\mathrm{C}\mathrm{O}}_{2}}$ (+ 31%; *P* = 0.014; g = -1.28, large; CI_95%_ = -0.34–2.19, panel E).

**Fig. 3:**
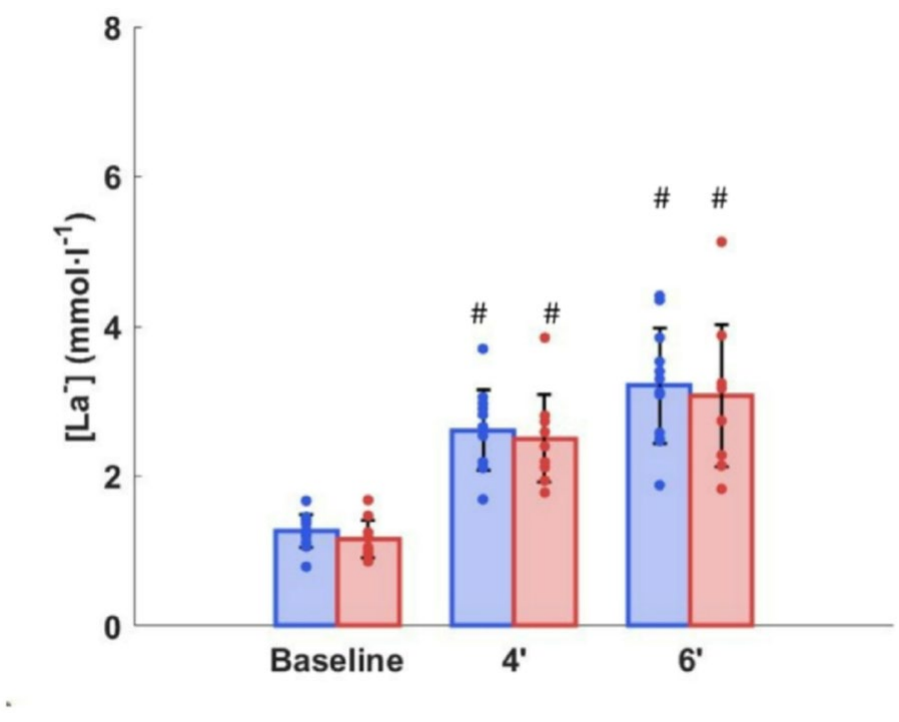
Blood lactate concentration [La^−^] at baseline and at the fourth and sixth minute of moderate-intensity square wave exercise in smokers (red bars) and controls (blue bars). Data are presented as mean ± standard deviation (SD); # *P* < 0.05 vs. baseline

Regarding [La^−^], a significant main effect of time was observed (F(2,14) = 30.619, *P* < 0.001, pη² = 0.81). No significant group effect was found (*P* = 0.92), while a significant Time × Group interaction emerged (F(2,14) = 15.33, *P* < 0.001, pη² = 0.69) (Fig. 3).

## Discussion

The present study aimed to determine the effect of CS on cardiopulmonary and gas-exchange kinetics during and after moderate-intensity exercise in young, physically active SMK without known lung or cardiovascular disease. In line with the experimental hypothesis, SMK exhibited slower cardiopulmonary and gas-exchange kinetics during both the on- and off- phase, as demonstrated by longer τ for $\:{f}_{\mathrm{H}}$, $\:\dot{\mathrm{Q}}$, $\:{\dot{\mathrm{V}}}_{{\mathrm{O}}_{2}}$, $\:{\dot{\mathrm{V}}}_{\mathrm{E}}$ and $\:{\dot{\mathrm{V}}}_{{\mathrm{C}\mathrm{O}}_{2}}$. These results suggest that the CS had already a negative impact on the parameters of the cardiopulmonary and gas-exchange kinetics even in young individuals with relatively short smoking history and without known lung or cardiovascular disease.

### Preliminary considerations

The lack of differences in static lung volumes between groups, recently discussed in a study by our group (Borrelli et al. 2025), is in line with prior research on young SMK (Lorensia et al. 2021). This finding might be attributed to the short smoking history of our SMK, which was not enough to produce airflow obstruction through bronchial remodelling, typically occurring only after prolonged CS exposure (Elbehairy et al. 2016; Melliti et al. 2021). In the present study, the reduced PEF could reflect a tendency towards to an increased airway resistance (Miller 2005), along with a decrease in MVV, unrelated to body size, suggesting diminished pulmonary endurance, which leads to dyspnoea and exercise limitation (Andrello et al. 2021; Miller et al. 2005; Pellegrino et al. 2005).

Since our study matched SMK and CTRL for age, anthropometric characteristics and physical activity levels, the observed lower $\:{\dot{\mathrm{V}}}_{{\mathrm{O}}_{2}\mathrm{m}\mathrm{a}\mathrm{x}}$ and $\:{\dot{\mathrm{W}}}_{\mathrm{m}\mathrm{a}\mathrm{x}}$ in SMK indicate that the benefits associated with regular aerobic training could be blunted by CS effects, confirming our previous findings (Borrelli et al. 2025). Explanation was given that the reduced $\:{\dot{\mathrm{V}}}_{{\mathrm{O}}_{2}\mathrm{m}\mathrm{a}\mathrm{x}}$ in SMK may be partly due to lower O_2_ carrying capacity associated with elevated HbCO levels and with the potentially earlier onset of skeletal muscle fatigue (Mendonca et al. 2011; Borrelli et al. 2025). In fact, skeletal muscle fibres function, especially at mitochondrial level could be impaired by the reactive oxygen species and other oxidants contained in cigarettes (Neves et al. 2016).

Lastly, SMK had a lower $\:{\dot{\mathrm{V}}}_{\mathrm{E}\:\mathrm{m}\mathrm{a}\mathrm{x}}$ mainly driven by a lower $\:{f}_{\mathrm{R}\:\mathrm{m}\mathrm{a}\mathrm{x}}$, which can be possibly considered as an early marker of lung parenchyma stiffness even though the relatively young age of the participants and their MIP and MEP values seem to exclude this explanation. An increased stiffness in the lung parenchyma in young SM (~ 7 year of smoking history), attributed to structural alterations in collagen fibers organization that impair the mechanical function of the lungs was reported in an autopsy study (Karimi and Razaghi 2018). However, based on the data available in our study, we cannot exclude the possibility that the reduced $\:{f}_{\mathrm{R}\:\mathrm{m}\mathrm{a}\mathrm{x}}$ was influenced by the lower $\:{\dot{\mathrm{W}}}_{\mathrm{m}\mathrm{a}\mathrm{x}}$ reached by SMK.

### Effect of CS on the kinetics during and after moderate exercise

In line with our hypothesis, SMK in this study showed slower on- and off-kinetics for all cardiopulmonary and gas-exchange variables. The slower $\:{f}_{\mathrm{H}}$ and $\:\dot{\mathrm{Q}}$ kinetics suggest that, despite a relatively brief smoking history, SMK may show signs of impaired vagal cardiac modulation (Bernaards et al. 2003). The reduced vagal control and impaired autonomic regulation made the cardiovascular response less efficient and slower, resulting in delayed kinetics. The slower $\:{f}_{\mathrm{H}}$ adjustment is responsible for increasing the time required to reach the steady state, thereby increasing the O_2_ deficit, the tissues’ demand during exercise, and the peripheral muscle fatigue (Almas et al. 2017). In other words, the slower cardiac response to exercise may be reflected in slower $\:{\dot{\mathrm{V}}}_{{\mathrm{O}}_{2}}$ kinetics that may be constrained by the bulk O_2_ delivery to the limbs, for which the $\:\dot{\mathrm{Q}}$ kinetics serves as an index (Adami et al. 2011).

The slower $\:{\dot{\mathrm{V}}}_{{\mathrm{O}}_{2}}$ kinetics in SMK during the recovery phase results likely in a larger O_2_ debt accumulation in SMK, reflecting a delayed restoration of energy stores in peripheral skeletal muscles. In support of this hypothesis, studies in mice exposed to CS have shown slower phosphocreatine recovery and decreased activity of oxidative enzymes such as citrate synthase and phosphofructokinase (Azevedo et al. 2021). Although the physiological mechanisms underlying the slower $\:{\dot{\mathrm{V}}}_{{\mathrm{O}}_{2}}$ kinetics during recovery are not completely understood, they appear to be related, at least in part, to the prolonged recovery of energy stores in the peripheral skeletal muscles (Ferretti 2015; Ferretti et al. 2022). In principle, an increased ATP utilization or a decline in ATP production efficiency may contribute to slow the $\:{\dot{\mathrm{V}}}_{{\mathrm{O}}_{2}}$ kinetics (Ferretti 2015; Ferretti et al. 2022). Additional factors may increase O_2_ debt. Among these, a reduction in skeletal muscle fibre size and in oxidative enzyme activity, a capillary regression and a shift towards glycolytic muscle fibre types have been demonstrated in studies on animal models exposed to CS. In particular, it has been demonstrated that CS exposure in mice involve TNFα mediated down regulation of PGC1α as a key step in vascular and myocyte dysfunction that are most evident in oxidative and glycolytic skeletal muscle (Tang et al. 2010). Further supporting this phenomenon is the absence of differences in $\:{\dot{\mathrm{V}}}_{{\mathrm{O}}_{2}}$ AMP, despite SMK exercising at a 20 W lower work rate that could reflect a difference in exercise efficiency between groups. The prolonged post exercise O_2_ demand might also depend on the slower $\:{\dot{\mathrm{V}}}_{\mathrm{E}}$ kinetics (Nery et al. 1982). Slower $\:{\dot{\mathrm{V}}}_{\mathrm{E}}$ kinetics during the off-phase have been linked to factors such as prolonged hypermetabolism and tachycardia post-exercise, likely resulting from an exercise-induced increase in venous CO_2_ (Chick et al. 1990). Indeed, hypercapnia is well known to elevate $\:{f}_{\mathrm{H}}$ and induce hyperpnea, the latter occurring to sustain an elevated ventilation rate necessary for the elimination of excess venous CO_2_. Slower $\:{\dot{\mathrm{V}}}_{\mathrm{E}}$ kinetics could result also from several factors, including an inability of the ventilatory system response kinetics to match the excess CO₂ load kinetics, the presence and severity of CO₂-induced acid-base imbalance, slower convective transport from the periphery to the lungs or within the pulmonary circulation, or reduced buffering capacity and rate (Oren et al. 1982; Casaburi et al. 1989).

### Limitations

This study presents some known limitations. First, measurements of plethysmographic lung volumes, lung diffusion, HbCO level, and the assessment of O_2_ extraction at the muscle level, which would offer a more thorough view of the integrated heart-lung-muscle system, are not included. Additionally, the sample was small and restricted to male participants. Lastly, physical activity level during the recruitment process was assessed by a self-reported questionnaire (i.e., IPAQ), which, though validated (Craig et al. 2003), depends on participants’ accuracy in reporting their physical habits.

## Conclusion

CS appears to affect negatively the cardiopulmonary and gas-exchange response to moderate exercise in young, physically active males without known lung or cardiovascular diseases. Indeed, despite their young age and fitness level, SMK exhibited slower cardiopulmonary and gas-exchange kinetics during both on- and off-phase at moderate-intensity exercise. Specifically, the slower $\:{\dot{\mathrm{V}}}_{{\mathrm{O}}_{2}}$ and *f*_H_ kinetics during both on- and off-phases suggest a lower cardiopulmonary fitness and an elevated risk of cardiovascular disease, bringing attention to the early CS-induced damage to the integrated heart-muscle-lung system and its manifestation during exercise. Noteworthy, traditional cardiopulmonary tests may fail to detect early CS-induced alterations in this population that became apparent only when transient responses were analysed. This study reinforces the evidence that no level of CS exposure can be considered risk-free, providing further support for the importance of quitting smoking and for preventing initiation from an early age.

## Data Availability

Data generated during and/or analysed during the current study are available as Supporting Information.

## Abbreviations

AMP: Amplitude of the response
ANOVA: Analysis of variance
CS: Cigarette smoking
CO: Carbon monoxide
COPD: Chronic obstructive pulmonary disease
CTRL: Controls
$$\:{f}_{\mathrm{H}}$$: Heart rate
HbCO: Carboxyhaemoglobin
IPAQ: International Physical Activity Questionnaire
[La^−^]: Blood lactate concentration
MEP: Maximal expiratory pressure
MIP: Maximal inspiratory pressure
$\:{\mathrm{P}\mathrm{e}\mathrm{t}\mathrm{C}\mathrm{O}}_{2}$: End-tidal carbon dioxide pressure
$\:{\mathrm{P}\mathrm{e}\mathrm{t}\mathrm{O}}_{2}$: End-tidal oxygen pressure
$$\:\dot{\mathrm{Q}}$$: Cardiac output
RER: Respiratory exchange ratio
RPE_GEN_: Rate of general perceived exertion
RPE_MUSC_: Rate of muscular perceived exertion
RPE_RESP_: Rate of respiratory perceived exertion
SMK: Smokers
τ: Tau
t_D_: Time delay
$\:{\dot{\mathrm{V}}}_{{\mathrm{C}\mathrm{O}}_{2}}$: Carbon dioxide output
$$\:{\dot{\mathrm{V}}}_{\mathrm{E}}$$: Expiratory ventilation
$\:{\dot{\mathrm{V}}}_{\mathrm{E}}/{\dot{\mathrm{V}}}_{{\mathrm{C}\mathrm{O}}_{2}}$: Ventilatory equivalent for carbon dioxide
$\:{\dot{\mathrm{V}}}_{\mathrm{E}}/{\dot{\mathrm{V}}}_{{\mathrm{O}}_{2}}$: Ventilatory equivalent for oxygen
$\:{\dot{\mathrm{V}}}_{{\mathrm{O}}_{2}}$: Pulmonary oxygen uptake
$\:{\dot{\mathrm{V}}}_{{\mathrm{O}}_{2}\mathrm{m}\mathrm{a}\mathrm{x}}$: Maximum pulmonary oxygen uptake
V_T_: Tidal volume
VT_1_: First ventilatory threshold
VT_2_: Second ventilatory threshold
$$\:\dot{\mathrm{W}}$$: Mechanical power
$\:{\dot{\mathrm{W}}}_{\mathrm{m}\mathrm{a}\mathrm{x}}$: Maximal mechanical aerobic power
$\:{\mathrm{Y}}_{0}$: Value of the cardiopulmonary variables before the transient
